# Microarray-based identification of genes associated with cancer progression and prognosis in hepatocellular carcinoma

**DOI:** 10.1186/s13046-016-0403-2

**Published:** 2016-08-27

**Authors:** Fuqiang Yin, Lipei Shu, Xia Liu, Ting Li, Tao Peng, Yueli Nan, Shu Li, Xiaoyun Zeng, Xiaoqiang Qiu

**Affiliations:** 1 Medical Scientific Research Centre, Guangxi Medical University, 22 Shuangyong Rd, Nanning, Guangxi 530021 People’s Republic of China; 2 Key Laboratory of High-Incidence-Tumor Prevention and Treatment (Guangxi Medical University), Ministry of Education, 22 Shuangyong Rd, Nanning, Guangxi 530021 People’s Republic of China; 3 The First Affiliated Hospital, Guangxi Medical University, 22 Shuangyong Rd, Nanning, Guangxi 530021 People’s Republic of China; 4 Centre for Translational Medicine, Guangxi Medical University, 22 Shuangyong Rd, Nanning, Guangxi 530021 People’s Republic of China; 5 School of Public Health, Guangxi Medical University, 22 Shuangyong Rd, Nanning, Guangxi 530021 People’s Republic of China

**Keywords:** Hepatocellular carcinoma, Microarray, Progression, Prognosis

## Abstract

**Background:**

Hepatocellular carcinoma (HCC) is the third leading cause of cancer-related deaths. The average survival and 5-year survival rates of HCC patients still remains poor. Thus, there is an urgent need to better understand the mechanisms of cancer progression in HCC and to identify useful biomarkers to predict prognosis.

**Methods:**

Public data portals including Oncomine, The Cancer Genome Atlas (TCGA) and Gene Expression Omnibus (GEO) profiles were used to retrieve the HCC-related microarrays and to identify potential genes contributed to cancer progression. Bioinformatics analyses including pathway enrichment, protein/gene interaction and text mining were used to explain the potential roles of the identified genes in HCC. Quantitative real-time polymerase chain reaction analysis and Western blotting were used to measure the expression of the targets. The data were analysed by SPSS 20.0 software.

**Results:**

We identified 80 genes that were significantly dysregulated in HCC according to four independent microarrays covering 386 cases of HCC and 327 normal liver tissues. Twenty genes were consistently and stably dysregulated in the four microarrays by at least 2-fold and detection of gene expression by RT-qPCR and western blotting showed consistent expression profiles in 11 HCC tissues compared with corresponding paracancerous tissues. Eleven of these 20 genes were associated with disease-free survival (DFS) or overall survival (OS) in a cohort of 157 HCC patients, and eight genes were associated with tumour pathologic PT, tumour stage or vital status. Potential roles of those 20 genes in regulation of HCC progression were predicted, primarily in association with metastasis. *INTS8* was specifically correlated with most clinical characteristics including DFS, OS, stage, metastasis, invasiveness, diagnosis, and age.

**Conclusion:**

The significantly dysregulated genes identified in this study were associated with cancer progression and prognosis in HCC, and might be potential therapeutic targets for HCC treatment or potential biomarkers for diagnosis and prognosis.

**Electronic supplementary material:**

The online version of this article (doi:10.1186/s13046-016-0403-2) contains supplementary material, which is available to authorized users.

## Background

Hepatocellular carcinoma (HCC) is the third leading cause of cancer-related deaths [1]. There are 750,000 new cases of HCC and nearly 700,000 deaths each year, making this a particularly lethal form of cancer [2]. Over the past decade major progress has been made in our understanding of the risk factors and molecular pathways driving liver carcinogenesis, and these advances have led to substantial opportunities for HCC prevention, surveillance, early diagnosis, prediction of prognosis, and therapy [1]. However, the average survival of HCC patients is normally between 6 and 20 months [3], and long-term prognosis is poor with reported 5-year survival rates ranging from 17 to 53 % [4]. Thus, there is an urgent need to better understand the mechanism of cancer progression and development in HCC and to identify useful biomarkers for diagnosis and prognosis.

High-throughput profiling technologies such as microarrays and, more recently, next-generation sequencing have become invaluable tools for biomedical research, and large amounts of data generated by those tools, including mRNA expression, DNA methylation, and microRNA expression, are collected in public archives such as the major public projects The Cancer Genome Atlas (TCGA) [5] and the International Cancer Genome Consortium [6], and the most prominent primary data archives, ArrayExpress [7], Gene Expression Omnibus (GEO) [8], Oncomine [9] and the databases of the International Nucleotide Sequence Database Collaboration [10]. The wide range of those databases, the various ways in which publicly archived gene expression data are being used in support of new studies, and reuse of these public data can be very powerful [11]. In particular, reusing of the data has the potential to predict treatment response and disease progression and was advantageous to develop precision therapies [12]. For example, based on data retrieved from Oncomine, TCGA, and GEO, Liu et al. identified several genes associated with ovarian cancer progression [13] and drug resistance [14]. In a similar manner, we identified that upregulation of E2F transcription factor 3 is associated with poor prognosis in HCC [15]. In the present study, using data of mRNA expression, DNA methylation, and clinical data retrieved from Oncomine, GEO, and the TCGA cohort, we identified a group of genes associated with cancer progression and prognosis in HCC.

## Methods

### Samples

All patients who underwent curative hepatectomy for primary HCC at the First Affiliated Hospital of Guangxi Medical University between March 2015 and September 2015 were eligible for inclusion in this study. Total of 11 HCCs and the matched paracancerous tissues were collected during surgery and stored in a liquid nitrogen tank until use for mRNA isolation and protein extraction. The study was endorsed by the Ethics Committee of Guangxi Medical University and was performed according to the Declaration of Helsinki, 2013 edition. All patients received an explanation of the aims of the study and signed informed consent.

### mRNA isolation and quantitative real-time polymerase chain reaction (RT-qPCR) analysis

Total RNA from 11 HCC and their matched paracancerous tissues was isolated using a miRNeasy Mini Kit (Qiagen, Hilden, Germany). RNA was quantified by spectrophotometry on a NanoDrop 2000 (Thermo Scientific, DE, USA). A total of 2 μg RNA was subjected to cDNA synthesis using the miScript II RT Kit (Qiagen, Hilden, Germany). RT-qPCR was performed with the QuantiFast SYBR Green PCR Kit (Qiagen, Hilden, Germany). Data were collected with the StepOnePlus Real-Time PCR System (ABI, CA, USA) according to the manufacturer’s instructions. The gene expression was compared in each HCC sample and the matched paracancerous tissue, and then the homogeneity of variance in all samples was analysed using the t-test. The RT-qPCR gene-specific primers were as follows: TBCE: forward primer, 5′-AGGCCAACAGATGTTCTCCAG-3′, reverse primer, 5′-CAGGGGGTTTCTTAGGCAGG-3′; INTS8: forward primer, 5′-AACTGAGAGTTCTACTGCTGGA-3′, reverse primer, 5′-GCTGCGCCCAAATCATAGC-3′; VIPR1: forward primer, 5′-TGCTGGGACACCATCAACTC-3′, reverse primer, 5′-TTGTCCGGAAAGAAGGCGAA-3′; CLEC4M: forward primer, 5′-TACTTCATGTCTAACTCCCAGCG-3′, reverse primer, 5′-GCTCCTCAGCAGTTTTGATTACG-3′; MARCO: forward primer, 5′-GGGGACACAGGACTTCAAGG-3′, reverse primer, 5′-CCCTGTTCTCCCTTCACACC-3′; DNASE1L3: forward primer, 5′-AGCCCTTTGTGGTCTGGTTC-3′, reverse primer, 5′-CGTCCGTGTAGACCTCAACC-3′; CRHBP: forward primer, 5′-AAATCCTCAGCAGGTTGCGA-3′, reverse primer, 5′-AAGGCGTCATCTTGGAAGGG-3′; FCN2: forward primer, 5′-CTGCAAGGACCTGCTAGACC-3′, reverse primer, 5′-TGTCATTCCCCAGCCAGAAC-3′; GAPDH (used as the control): forward primer, 5′-GAAGGTGAAGGTCGGAGT-3′, reverse primer, 5′-GAAGATGGTGATGGGATTT-3′.

### Protein extraction and western blotting

Total protein was extracted from HCC and paracancerous tissues with RIPA lysis buffer (Solarbio, Beijing, China) and proteinconcentration was determined using an Enhanced BCA Protein Quantification Kit (KeyGEN BioTECH, Jiangsu, China). Then the samples were separated by Novex NuPAGE SDS-PAGE Gel System (Thermo Fisher Scientific, MA, USA) and were transferred to the PVDF membrane using the Bio-Rad Criterion System (Bio-Rad, CA, USA). Membranes were blocked with 8 % non-fat dry milk in PBS containing 0.1 % Tween-20 (0.1 % TBST, pH7.4) for 1 h. Membranes were incubated with antibodies specific for human INTS8 (rabbit polyclonal antibody, 1:750 dilutions; Proteintech, Hubei, China) and GAPDH (rabbit polyclonal antibody, 1:1,000 dilution; Boster, Hubei, China) overnight at 4 °C. After 3 washings with 0.1 % TBST for 5 min, horseradish peroxidase-conjugated goat anti-rabbit secondary antibodies (1:5,000 dilution; Bioss, Beijing, China) were applied, followed by washings with 0.1 % TBST for 5 min each at room temperature (RT). The bound immunocomplexes were detected using ECL+ reagent (GE Healthcare Bio-Sciences, NJ, USA) with a FluorChem M system (Proteinsimple, CA, USA).

### Gene expression profiles

The genes significantly dysregulated in HCC were identified based on the 4 microarrays, Chen Liver microarray (104 HCCs vs. 76 liver tissues), Roessler Liver microarray (22 HCCs vs. 21 liver tissues), Roessler Liver 2 microarray (225 HCCs vs. 220 liver tissues) and Wurmbach Liver microarray (35 HCCs vs. 10 liver tissues), which are all deposited in Oncomine database (https://www.oncomine.org/resource/login.html) [9]. The 4 microarrays together covering total of 386 cases of HCCs and 327 cases of normal liver tissues. The rank for a gene is the median rank for that gene across each of the analyses. DNA methylation, mRNA expression, and clinical data of 379 HCC patients in a TCGA cohort were retrieved from cBioPortal for Cancer Genomics (http://cbioportal.org) [16, 17], but only 157 samples with matched gene expression data, prognosis data and most of the other clinical data were used to analyze the clinical importance of the target genes. mRNA expression data associated with HCC metastasis were retrieved from microarray GDS3091 [18] and GDS274 [19], which were deposited in the GEO profiles databases (http://www.ncbi.nlm.nih.gov/geoprofiles/) [8].

### Bioinformatics analyses

Enrichment of the biological process and cellular component of a group of genes was determined using the DAVID online tool (http://david.abcc.ncifcrf.gov/) [20, 21]. Protein/gene-protein/gene interaction analysis was performed using the GeneMANIA online tool (http://www.genemania.org/) [22, 23]. Function prediction based on text mining was performed using the Coremine Medical online database (http://www.coremine.com/medical/) [24].

### Data analysis

The data were analysed by SPSS 20.0 software. The mRNA expression of a gene is presented as the mean ± SD. Homogeneity of variance was analysed using the t-test. Expression values of a gene were dichotomised into high and low expression using the median as a cutoff for analysis of clinical importance in a TCGA cohort, as described in a previous study [25]. The probability of survival and its significance was calculated using the Kaplan-Meier method and log-rank test, respectively. A Cox proportional hazard model was performed for multivariate analysis of prognosis. The correlation between gene expression and clinicopathologic characteristics was evaluated by Pearson’s χ^2^ test (two-sided). The correlation between DNA methylation and gene expression was analysed using bivariate correlations. *P* values < 0.05 were considered to indicate statistically significant differences.

## Results

### Retrieval of significantly dysregulated genes in HCC

**Fig. 1:**
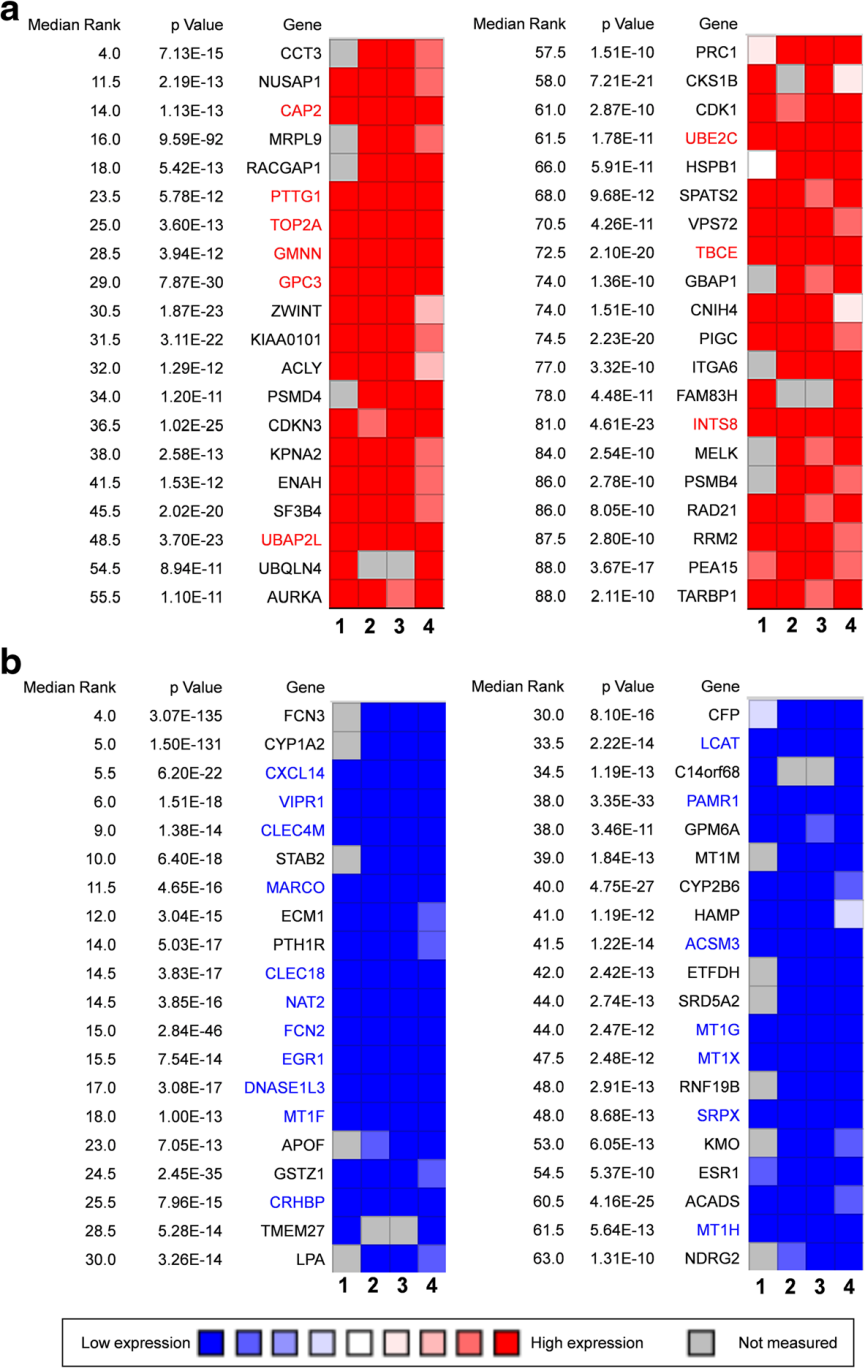
The 80 genes that were significantly dysregulated in hepatocellular carcinomas according to four independent microarrays retrieved from the Oncomine database. **a** The top 40 genes that were significantly upregulated in four microarrays. **b** The top 40 genes that were significantly downregulated in four microarrays. The four microarrays cover a total of 386 cases of hepatocellular carcinomas and 327 cases of normal liver tissue: (1) Chen Liver Statistics, 104 cases of hepatocellular carcinoma and 76 cases of liver tissue; (2) Roessler Liver Statistics, 22 cases of hepatocellular carcinoma and 21 cases of liver tissue; (3) Roessler Liver 2 Statistics, 225 cases of hepatocellular carcinoma and 220 cases of liver tissue; (4) Wurmbach Liver Statistics, 35 cases of hepatocellular carcinoma and 10 cases of liver tissue. The rank for a gene is the median rank for that gene across each of the analyses. The *P* value given for a gene is for the median-ranked analysis. The genes labelled in *red* and in *blue* were significantly and consistently up- and downregulated in the four microarrays, respectively

Four independent microarrays deposited in the Oncomine database were selected to identify genes associated with cancer development and progression in HCC. These microarrays were Chen Liver Statistics covering 104 cases of HCC and 76 cases of liver tissue, Roessler Liver Statistics covering 22 cases of HCC and 21 cases of liver tissue, Roessler Liver 2 Statistics covering 225 cases of HCC and 220 cases of liver tissue, and Wurmbach Liver Statistics covering 35 cases of HCC and 10 cases of liver tissues. Based on analysis of these four independent microarrays, 40 genes that were significantly upregulated (*P* < 1.36E-10) and 40 genes that were significantly downregulated (*P* < 1.31E-10) in HCC were retrieved (Fig. 1). Analysis of the 80 genes by the DAVID online tool indicated that cell cycle was the top biological process, covering 17 genes, and microtubule cytoskeleton was the top cellular component, covering 14 genes (Additional file 1: Table S1).

Among the 80 genes that were dysregulated in HCCs according to four independent microarrays covering a total of 386 cases of HCC and 327 cases of normal liver tissues, nine genes (*CAP2*, *PTTG1*, *TOP2A*, *GMNN*, *GPC3*, *UBE2C*, *UBAP2L*, *TBCE*, and *INTS8*) were consistently and stably upregulated and 18 genes (*CXCL14*, *VIPR1*, *CLEC4M*, *MARCO*, *CLEC1B*, *NAT2*, *FCN2*, *EGR1*, *DNASE1L3*, *MT1F*, *CRHBP*, *LCAT*, *PAMR1*, *ACSM3*, *MT1G*, *MT1X*, *SRPX*, and *MT1H*) were consistently and stably downregulated in HCC, by least 2-fold (Fig. 1; Table 1). Among the above 27 genes, seven genes—*CAP2*, *GMNN*, *PTTG1*, *TBCE*, *TOP2A*, *UBE2C*, and *FCN2*—encode proteins associated with cell cycle and microtubule cytoskeleton (Additional file 1: Table S1). Protein/gene-protein/gene interaction analysis was performed to further explain the interrelationships of these genes in HCC. As shown in Additional file 2: Figure S2, the 27 proteins/genes directly/indirectly interacted with each other via co-localisation, genetic interactions, shared common pathways, and protein domains, and, in particular, co-expression, and 10 of them—*VIPR1*, *DNASE1L3*, *SRPX*, *MT1H*, *CXCL14*, *CLEC4M*, *CRHBP*, *GPC3*, *NAT2*, and *MARCO*—interacted with at least 14 other genes, more than half of all the genes in the interaction network (Additional file 2: Figure S2). Moreover, these genes were also those that were dysregulated at least 4-fold in HCC (Table 1).

**Table 1:** Genes that were stably and consistently dysregulated in 386 cases of hepatocellular carcinoma compared with 327 cases of normal liver tissues according to four independent microarrays retrieved from the Oncomine database, and their associations with hepatocellular carcinoma

Gene	Independent microarray data (Fold change)					No. of articles^a^	Associations with hepatocellular carcinoma
	Direction of regulation	Chen Liver	Roessler Liver	Roessler Liver 2	Wurmbach Liver		
*TBCE* ^b^	Up	2.125	2.403	2.822	2.419	-	-
*INTS8* ^b^	Up	2.393	3.102	2.340	2.115	-	-
*UBAP2L* ^b^	Up	2.108	2.959	2.819	2.742	-	-
*GMNN* ^b^	Up	3.362	7.340	4.696	3.394	1	Potential oncogene [38]
*UBE2C*	Up	4.733	3.661	3.422	5.113	4	Cancer progression and poor prognosis [39]
*PTTG1*	Up	4.688	4.741	5.773	10.622	9	Angiogenesis, progression, and poor prognosis [40, 41], therapeutic target [42]
*CAP2*	Up	3.526	4.254	5.790	8.569	10	Multistage hepatocarcinogenesis [43], early detection [44]
*TOP2A*	Up	2.663	11.236	8.292	13.321	11	Early age onset, shorter patient survival and chemoresistance [45]
*GPC3*	Up	16.826	26.693	28.236	76.162	199	Diagnosis [29], cell proliferation and invasion [28]; prediction of recurrence [30]
*VIPR1* ^b^	Down	9.979	5.310	7.202	4.855	-	-
*CLEC4M* ^b^	Down	28.107	9.276	4.361	36.431	-	-
*MARCO* ^b^	Down	11.333	6.107	3.984	20.154	-	-
*DNASE1L3* ^b^	Down	8.386	12.378	7.653	10.303	-	-
*PAMR1* ^b^	Down	2.726	2.381	2.473	2.917	-	-
*ACSM3* ^b^	Down	2.902	6.135	4.836	11.262	-	-
*CLEC1B* ^b^	Down	6.600	6.605	4.748	36.770	1	Downregulated in a cohort of 65 pairs of human HCCs [46]
*MT1F* ^b^	Down	14.107	18.140	15.749	9.680	1	Inhibition of cancer growth [47]
*CRHBP* ^b^	Down	16.565	7.020	4.822	46.837	1	Downregulated in a cohort of 65 pairs of human HCCs [46]
*LCAT* ^b^	Down	4.917	8.507	8.064	7.435	1	LCAT activity correlated with serum albumin and serum bilirubin level [48]
*MT1X* ^b^	Down	10.812	11.558	8.227	6.903	1	HCC-related [49]
*SRPX* ^b^	Down	4.929	5.104	5.879	7.202	1	Proliferation, migration and invasiveness [50]
*MT1H* ^b^	Down	13.846	9.037	8.473	7.723	1	Potential tumour suppressor [51]
*FCN2* ^b^	Down	10.881	9.089	6.299	44.688	2	HBV- and HCV-related HCC [52], FCN2 haplotypes associate with HCC [53]
*CXCL14* ^b^	Down	12.903	9.667	10.940	13.977	4	Potential diagnostic marker [54]; rs2237062 polymorphism influences HBV-related HCC progression [52, 55]; potential tumour suppressor [56]
*MT1G* ^b^	Down	13.065	11.134	11.160	11.187	4	Tumour suppressor gene [51, 57], biomarker [58]
*EGR1*	Down	3.541	10.547	6.769	9.241	12	Critical for hepatocarcinogenesis [59]
*NAT2*	Down	8.024	16.088	13.999	36.890	14	NAT2 polymorphism is risk factor for developing HCC [60], NAT2 activity is critical in smoking-related hepatocarcinogenesis [61]

^a^No. of articles was based on a search in the PubMed database

^b^poorly studied genes in HCC

### Measurement of gene expression at mRNA and protein level

**Fig. 2:**
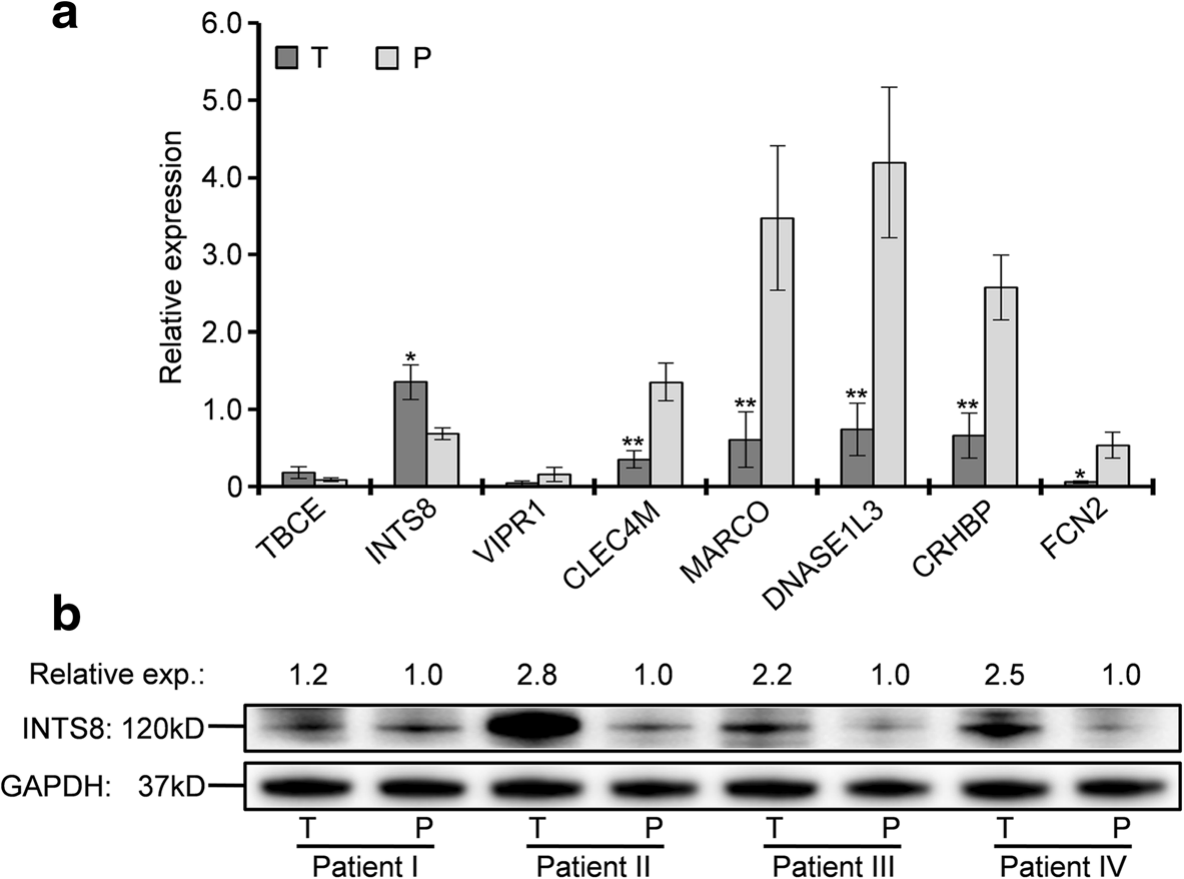
Measurement of gene expression at mRNA and protein level. **a** mRNA expression of genes in 11 tissues of HCC patients compared with matched paracancerous tissue. * *P* < 0.05; ** *P* < 0.01. **b** Protein expression of INTS8 in four tissues of HCC patients compared with expression in corresponding paracancerous tissues. The intensity of protein bands was measured by Image J software.. T, HCC tissue; P, paracancerous tissue

Among the 27 genes, the associations of seven with HCC are relatively well studied and described in published papers. However, the relationship of the remaining 20 genes with HCC was poorly understood, and these genes were selected for further analyses (Table 1). The expression of eight genes that were randomly selected from the 20 genes was measured by RT-qPCR in 11 tissues of HCC patients compared with matched paracancerous tissues. As shown in Fig. 2a, the expression of *TBCE* and *INTS8* was increased, whereas that of *VIPR1*, *CLEC4M*, *MARCO*, *DNASE1L3*, *CRHBP*, and *FCN2* was decreased in HCC tissues, although the changes in *TBCE* and *VIPR1* expression were not statistically significant. Compared with the average expression in paracancerous tissues, the expression of *INTS8* in HCC was upregulated with 2.06-fold and the expression of *CLEC4M*, *MARCO*, *DNASE1L3*, *CRHBP*, and *FCN2* was downregulated with 3.83-, 5.70-, 5.63-, 3.87-, and 8.94-fold, respectively. All results of gene expression determined by RT-qPCR were completely consistent with their expression identified by the four independent microarrays (Fig. 1; Table 1). Furthermore, a significant increase at the protein level of INTS8 was observed in HCC tissues compared with corresponding paracancerous tissues (Fig. 2b), which was consistent with its expression at the mRNA level.

### Analysis of clinical importance

The clinical importance in HCC of the 20 selected genes (Table 1) was evaluated on the basis of TCGA clinical data. A total of 379 HCC patient samples with clinical data in a cohort of TCGA were retrieved. Among these, 157 samples with mRNA expression values were selected for analysis of the relationship between genes and clinical characteristics. The expression values of a gene were categorised as high or low according to the median value in accordance with a previous study [25].

**Fig. 3:**
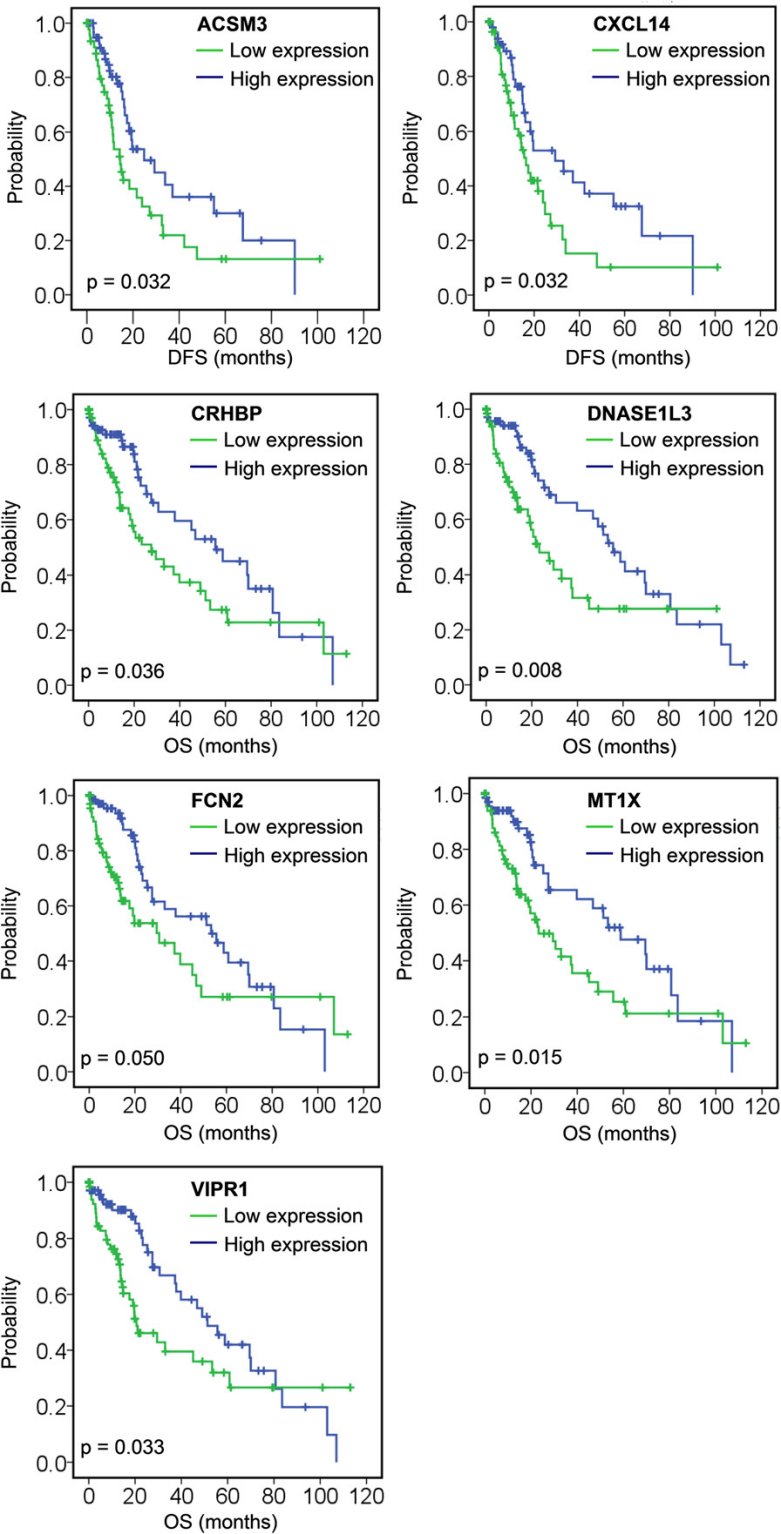
Association of seven genes (*ACSM3*, *CXCL14*, *CRHBP*, *DNASE1L3*, *FCN2*, *MT1X*, and *VIPR1*) with DFS or OS, analysed using Kaplan-Meier survival plots. The survival data of 157 HCC patients in a TCGA cohort were used for the analysis. Expression values of a gene were dichotomised into high expression (*blue* line) and low expression (*green* line) using the median as a cutoff

**Fig. 4:**
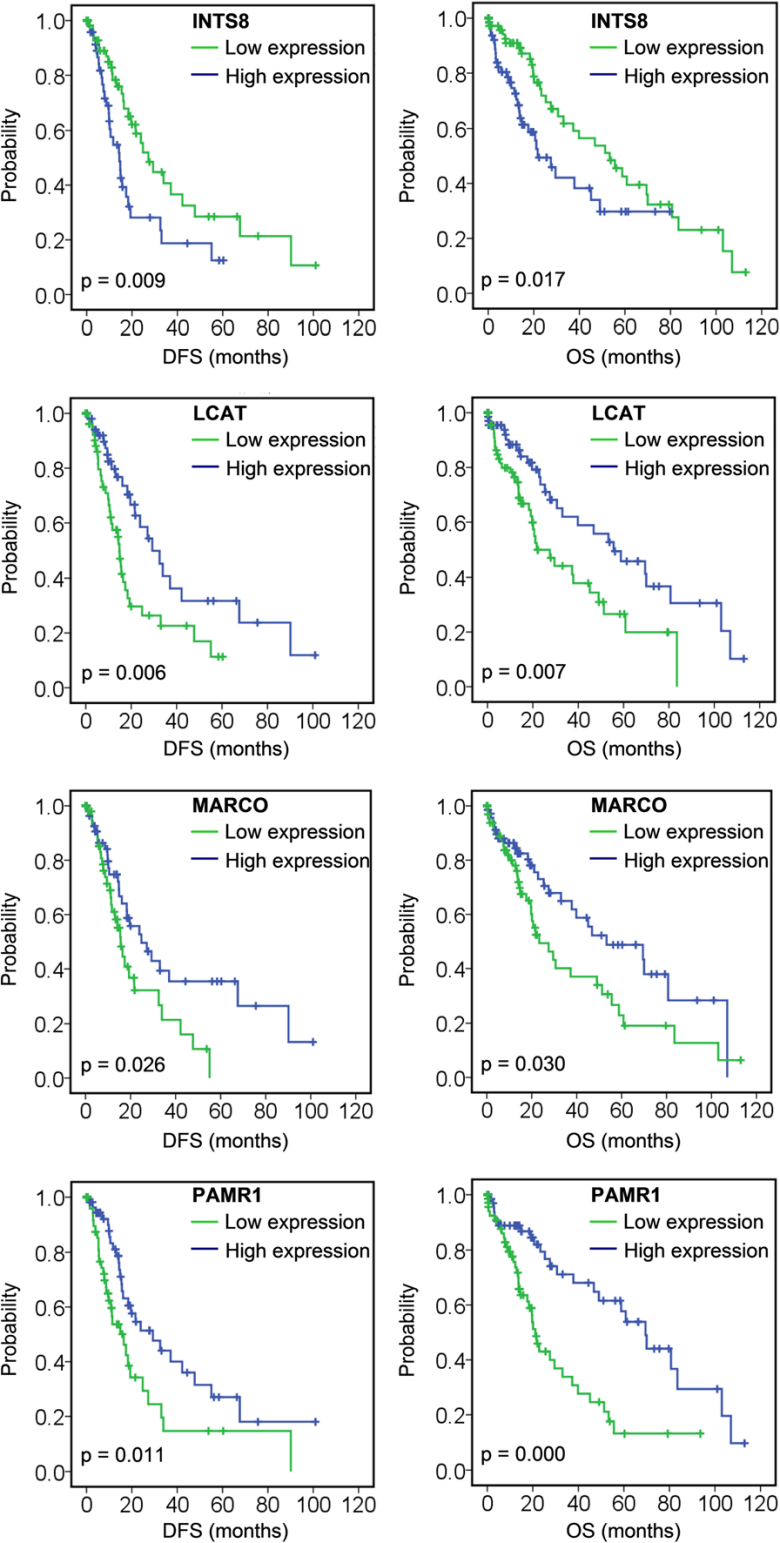
Association of *INTS8*, *LCAT*, *MARCO*, and *PAMR1* with DFS and OS, analysed using Kaplan-Meier survival plots. The survival data of 157 HCC patients in a TCGA cohort were used for the analysis. Expression values of a gene were dichotomised into high expression (*blue* line) and low expression (*green* line) using the median as a cutoff

A total of 11 genes were associated with DFS and/or OS (Table 2); among those, low expression of *ACSM3* and *CXCL14* was associated with poor DFS, and low expression of *CRHBP*, *DNASE1L3*, *FCN2*, *MT1X*, and *VIPR1* was associated with poor OS (Fig. 3, Table 2). Four genes were associated with both DFS and OS: high expression of *INTS8* in HCC patients, and low expression of *LCAT*, *MARCO*, and *PAMR1*, was associated with poor DFS and OS (Fig. 4, Table 2). To elucidate whether any of the above genes was an independent factor for predicting patient survival, we performed multivariate analyses of tumour stage, tumour pathologic PT, tumour residual, tumour status, vital status, age, gender, and the 11 genes by a Cox proportional hazards model (Table 3). We found that stage (*P* = 0.050), tumour status (*P* = 0.001), *DNASE1L3* expression (*P* = 0.042), and *INTS8* expression (*P* = 0.023) were independent risk prognostic factors for OS in HCC patients, although no gene was found to be an independent prognostic factor for DFS (data not shown).

**Table 2:** The associations of 11 genes with disease-free survival (DFS) and/or overall survival (OS) of patients with hepatocellular carcinoma in a TCGA cohort, analysed using Kaplan-Meier survival plots

		DFS (Median)				OS (Median)			
				95 % Confidence Interval				95 % Confidence Interval	
		Estimate	Std. Error	Lower Boundary	Upper Boundary	Estimate	Std. Error	Lower Boundary	Upper Boundary
ACSM3	H	24.800	7.587	9.930	39.670				
	L	14.400	2.391	9.714	19.086				
	Overall	19.300	3.428	12.581	26.019				
CXCL14	H	29.300	10.355	9.004	49.596				
	L	16.400	2.222	12.045	20.755				
	Overall	19.300	3.428	12.581	26.019				
INTS8	H	14.400	2.408	9.681	19.119	21.700	5.048	11.805	31.595
	L	27.200	4.266	18.839	35.561	53.300	10.531	32.659	73.941
	Overall	19.300	3.428	12.581	26.019	37.800	8.792	20.568	55.032
LCAT	H	29.300	5.287	18.937	39.663	55.600	13.029	30.063	81.137
	L	14.800	2.129	10.628	18.972	21.700	5.133	11.640	31.760
	Overall	19.300	3.428	12.581	26.019	37.800	8.792	20.568	55.032
MARCO	H	24.800	6.094	12.856	36.744	53.300	16.525	20.911	85.689
	L	15.600	1.710	12.248	18.952	23.300	5.664	12.199	34.401
	Overall	19.300	3.428	12.581	26.019	37.800	8.792	20.568	55.032
PAMR1	H	29.300	7.881	13.853	44.747	69.500	7.445	54.908	84.092
	L	16.400	3.616	9.312	23.488	21.100	1.762	17.647	24.553
	Overall	19.300	3.428	12.581	26.019	37.800	8.792	20.568	55.032
CRHBP	H					55.600	13.080	29.964	81.236
	L					27.500	7.523	12.754	42.246
	Overall					37.800	8.792	20.568	55.032
DNASE1L3	H					55.600	6.310	43.232	67.968
	L					23.300	5.103	13.298	33.302
	Overall					37.800	8.792	20.568	55.032
FCN2	H					53.300	12.677	28.453	78.147
	L					30.600	10.341	10.331	50.869
	Overall					37.800	8.792	20.568	55.032
MT1X	H					58.800	12.301	34.690	82.910
	L					23.300	5.997	11.546	35.054
	Overall					37.800	8.792	20.568	55.032
VIPR1	H					51.300	7.615	36.374	66.226
	L					20.600	5.643	9.540	31.660
	Overall					37.800	8.792	20.568	55.032

The gene expression and survival data of 157 HCC patients in a TCGA cohort were used for the analysis. Expression values of a gene were dichotomised into high and low expression using the median as a cutoff

*H* high expression, *L* low expression

**Table 3:** Multivariate analysis of prognosis of 157 HCC patients in a TCGA cohort using Cox proportional hazard model

Factors	B	SE	Wald	df	Sig.	Exp(B)	95.0 % CI	
							Lower	Upper
*CRHBP*	-.267	1.272	.044	1	.834	.766	.063	9.268
*DNASE1L3*	-.969	.476	4.140	1	.042	.379	.149	.965
*FCN2*	.517	.896	.333	1	.564	1.676	.290	9.704
*INTS8*	.204	.090	5.175	1	.023	1.227	1.029	1.463
*LCAT*	.030	.194	.024	1	.877	1.031	.704	1.509
*MARCO*	-.070	.859	.007	1	.935	.932	.173	5.020
*MT1X*	.788	1.051	.561	1	.454	2.198	.280	17.256
*PAMR1*	-.158	.236	.448	1	.503	.854	.538	1.355
*VIPR1*	.194	.287	.459	1	.498	1.215	.692	2.131
Stage (I/II–III)	.901	.463	3.784	1	.050	2.462	.993	6.101
PT (1–2/3–4)	-.223	.426	.273	1	.601	.800	.347	1.844
Residual (R0/R1–2)	-.175	.586	.089	1	.765	.839	.266	2.649
Tumour status (free/with)	1.300	.404	10.359	1	.001	3.669	1.662	8.097
Vital status (dead/alive)	−13.599	55.193	.061	1	.805	.000	.000	1.2e + 41
Age	.000	.014	.001	1	.981	1.000	.974	1.028
Gender	-.276	.312	.781	1	.377	.759	.412	1.399

*PT* AJCC Tumour Pathologic PT

Six genes were associated with tumour pathologic PT and tumour stage (Table 4); among these, high expression of *INTS8* and *UBAP2L*, and low expression of *ACSM3*, *FCN2*, *LCAT*, and *MT1G*, was significantly associated with metastatic tumour and late stage (*P* ≤ 0.05). In particular, *UBAP2L* was markedly and highly expressed in T2 tumours (72.5 % vs. 27.5 %) and *LCAT* was lowly expressed in T2 tumours (30.0 % vs. 70.0 %) and highly expressed in T1 tumours (72.6 % vs. 27.4 %). In addition, *LCAT* was highly expressed in stage I tumours (71.2 % vs. 28.8 %).

**Table 4:** Associations of genes expression with AJCC tumour pathologic PT, tumour stage, age and gender in 157 patients with hepatocellular carcinoma

Factors	No. of patients	*ACSM3*		*FCN2*		*INTS8*		*LCAT*		*MT1G*		*UBAP2L*	
		High	Low	High	Low	High	Low	High	Low	High	Low	High	Low
PT	157	*P* = 0.037		*P* = 0.026		*P* = 0.046		*P* = 0.000		*P* = 0.016		*P* = 0.004	
T1	62 (39.5 %)	39 (62.9 %)	23 (37.1 %)	39 (62.9 %)	23 (37.1 %)	23 (37.1 %)	39 (62.9 %)	45 (72.6 %)	17 (27.4 %)	39 (62.9 %)	23 (37.1 %)	22 (35.5 %)	40 (64.5 %)
T2	40 (25.5 %)	20 (50.0 %)	20 (50.0 %)	13 (32.5 %)	27 (67.5 %)	24 (60.0 %)	16 (40.0 %)	12 (30.0 %)	28 (70.0 %)	13 (32.5 %)	27 (67.5 %)	29 (72.5 %)	11 (27.5 %)
T3	46 (29.3 %)	16 (34.8 %)	30 (65.2 %)	22 (47.8 %)	24 52.2 %)	28 (60.9 %)	18 (39.1 %)	18 (39.1 %)	28 (60.9 %)	21 (45.7 %)	25 54.3 %)	23 (50.0 %)	23 (50.0 %)
T4	9 (5.7 %)	4 (44.4 %)	5 (55.6 %)	4 (44.4 %)	5 (55.6 %)	4 (44.4 %)	5 (55.6 %)	4 (44.4 %)	5 (55.6 %)	6 (66.7 %)	3 (33.3 %)	5 (55.6 %)	4 (44.4 %)
Stage	143	*P* = 0.016		*P* = 0.032		*P* = 0.026		*P* = 0.000		*P* = 0.037		*P* = 0.009	
I	59 (41.3 %)	36 (61.0 %)	23 (39.0 %)	36 (61.0 %)	23 (39.0 %)	23 (39.0 %)	36 (61.0 %)	42 (71.2 %)	17 (28.8 %)	37 (62.7 %)	22 (37.3 %)	22 (37.3 %)	37 (62.7 %)
II	36 (25.2 %)	19 (52.8 %)	17 (47.2 %)	12 (33.3 %)	24 (66.7 %)	22 (61.1 %)	14 (38.9 %)	12 (33.3 %)	24 (66.7 %)	13 (36.1 %)	23 (63.9 %)	25 (69.4 %)	11 (30.6 %)
III	48 (33.6 %)	16 (33.3 %)	32 66.7 %)	25 (52.1 %)	23 (47.9 %)	30 (62.5 %)	18 (37.5 %)	18 (37.5 %)	30 (62.5 %)	23 (47.9 %)	25 (52.1 %)	25 (52.1 %)	23 (47.9 %)
		*CXCL14*		*GMNN*		*INTS8*		*MT1F*		*MT1G*		*SPRX*	
		High	Low	High	Low	High	Low	High	Low	High	Low	High	Low
Age^a^	157	*P* = 0.031		*P* = 0.031		*P* = 0.005		*P* = 0.013		*P* = 0.031		*P* = 0.031	
< 65	80 (51.0 %)	47 (58.8 %)	33 (41.3 %)	47 (58.8 %)	33 (41.3 %)	49 (61.2 %)	31 (38.8 %)	47 (58.8 %)	32 (40.0 %)	47 (58.8 %)	33 (41.3 %)	47 (58.8 %)	33 (41.3 %)
≥ 65	77 (49.0 %)	32 (41.6 %)	45 (58.4 %)	32 (41.6 %)	45 (58.4 %)	30 (39.0 %)	47 (61.0 %)	32 (41.6 %)	46 (59.7 %)	32 (41.6 %)	45 (58.4 %)	32 (41.6 %)	45 (58.4 %)
		*CLEC1B*		*CRHBP*		*FCN2*		*MT1G*		*TBCE*			
		High	Low	High	Low	High	Low	High	Low	High	Low		
Gender	157	*P* = 0.003		*P* = 0.019		*P* = 0.043		*P* = 0.003		*P* = 0.019			
Female	62 (39.5 %)	22 (35.5 %)	40 (64.5 %)	24 (38.7 %)	38 (61.3 %)	25 (61.0 %)	37 (39.0 %)	22 (35.5 %)	40 (64.5 %)	24 (38.7 %)	38 (61.3 %)		
Male	95 (60.5 %)	57 (60.0 %)	38 (40.0 %)	55 (57.9 %)	40 (42.1 %)	54 (56.8 %)	41 (43.2 %)	57 (60.0 %)	38 (40.0 %)	55 (57.9 %)	40 (42.1 %)		

^a^Age was dichotomised into < 65 and ≥ 65 using the median as a cutoff. PT, AJCC Tumour Pathologic PT. Expression values of a gene were dichotomised into high and low expression using the median as a cutoff. P value determined using Pearson’s χ^2^ test (2-sided)

Ten genes were associated with age and gender. As shown in Table 4, we found that six genes—*CXCL14*, *GMNN*, *INTS8*, *MT1F*, *MT1G*, and *SPRX*—were expressed at low levels in HCC patients aged ≥ 65 years. Expression of five genes was related to the gender of HCC patients. Except for *FCN2*, which is lowly expressed in male HCC patients, the other four genes, *CLEC1B*, *CRHBP*, *MT1G*, and *TBCE*, were all lowly expressed in female HCC patients. In addition, *PAMR1* and *MT1X* were closely related to the vital status; both showed low expression in 60.3 % (38/63) of HCC patients with dead status, compared with high expression in 57.4 % (54/94) of patients with alive status (*P* = 0.022).

### Potential roles of the genes in HCC progression

**Fig. 5:**
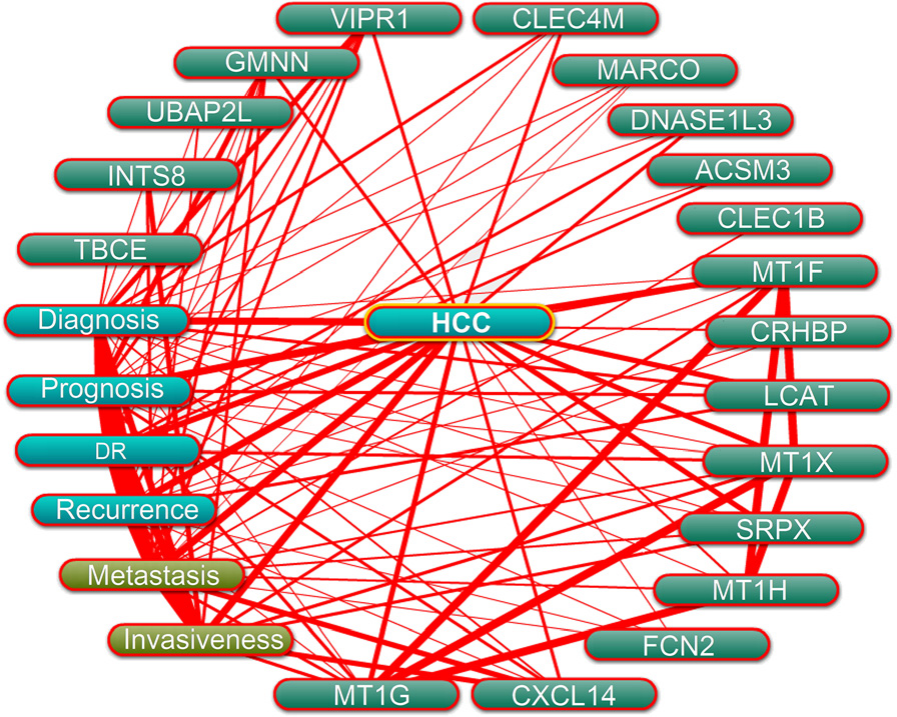
Association of the genes with HCC characteristics was determined by text mining using Coremine Medical and probabilistic scoring (*P* < 0.05). HCC: hepatocellular carcinoma, DR: drug resistance

The potential roles of the 20 genes in HCC were predicted on the basis of Coremine Medical mining. As shown in Fig. 5, the associations of the genes with diagnosis, prognosis, drug resistance, recurrence, metastasis, and invasiveness of HCC was comprehensively analysed. The results indicated that, with the exception of *PAMR1*, the other 19 genes were all associated with at least one factor contributing to cancer progression, and many of the genes, for example *GMNN*, *CXCL14*, *MT1G*, *MT1X*, *SPRX*, and *VIPR1*, were closely associated with almost all of the factors included in this analysis. Most of the genes were extensively associated with several factors. For example, 15 genes (including *INTS8*, *LCAT*, *MARCO*, and *DANSE1L3*) were associated with diagnosis, 14 genes (including *INTS8*, *MARCO*, *CRHBP*, and *VIPR1*) were associated with metastasis, and 13 genes (including *LCAT*, *MARCO*, *FCN2*, and *CXCL14*) were associated with prognosis.

**Fig. 6:**
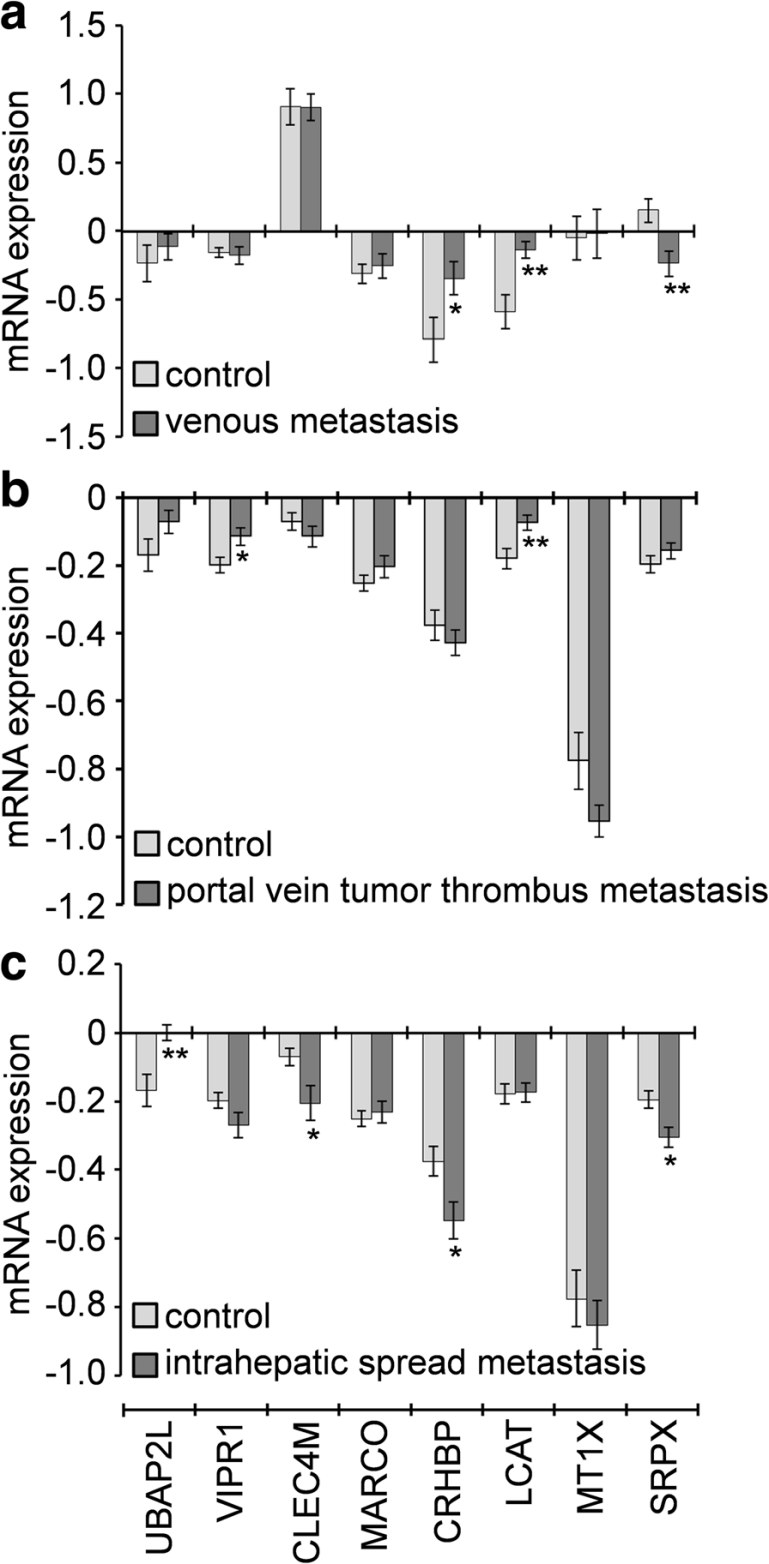
mRNA expression of the genes in HCC patients with and without metastasis according to microarray data retrieved from the GEO online database. **a** Microarray data GDS3091 [18] cover nine HCCs with venous metastasis and 11 without as controls. **b**, **c** Microarray data GDS274 [19] cover 32 HCCs with portal vein tumour thrombus metastasis, 33 with intrahepatic spread metastasis, and 22 HCCs with no metastasis as controls. *, *P* < 0.05; **, *P* < 0.01

Based on the gene expression in two independent GEO microarrays corresponding to HCC metastasis, the association of the genes *CLEC4M*, *CRHBP*, *MARCO*, *MT1X*, *SRPX*, *UBAP2L*, and *VIPR1* with metastasis was further analysed; unfortunately, data for the other genes were unavailable. The expression of *CRHBP*, *LCAT*, and *SPRX* was significantly dysregulated in nine HCCs with venous metastasis compared with 11 HCC without (Fig. 6a). Genes *VIPR1*, *LCAT*, *BAP2L*, *CLEC4M*, *CRHBP*, and *SRPX* were significantly dysregulated in 32 HCCs with portal vein tumour thrombus metastasis and 33 HCCs with intrahepatic spread metastasis compared with 22 HCCs with no metastasis (Fig. 6b&c). In particular, *LCAT* was highly expressed in HCC patients with venous metastasis and patients with portal vein tumour thrombus metastasis, and *SRPX* was lowly expressed in HCC patients with venous metastasis and patients with intrahepatic spread metastasis (Fig. 6).

### Correlation of DNA methylation with mRNA expression of the target genes

**Fig. 7:**
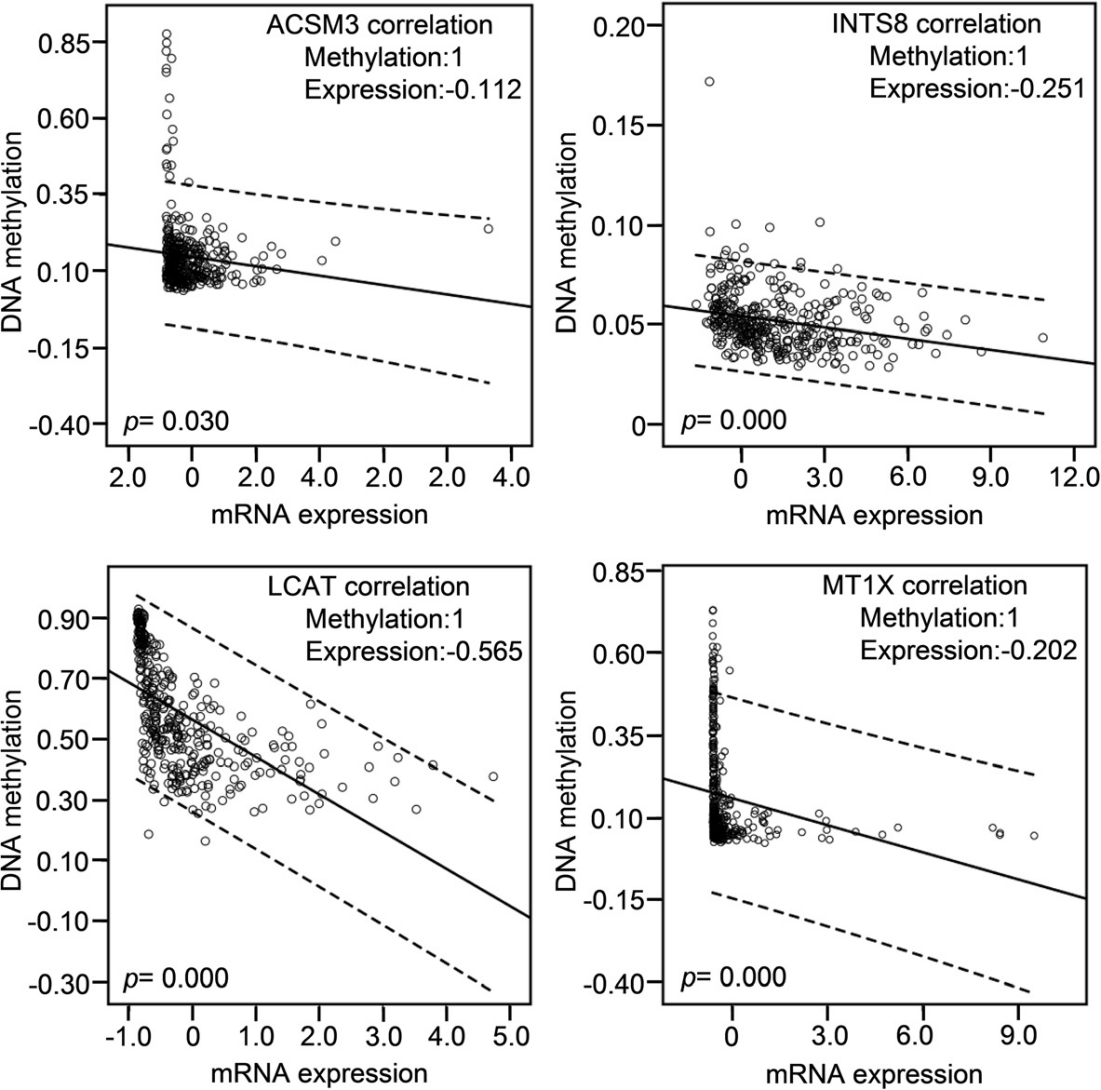
DNA methylation of four genes was significantly and negatively correlated with their mRNA expression. Data for gene expression and DNA methylation in 379 HCCs were retrieved from a TCGA cohort. The correlation between DNA methylation and gene expression was analysed using bivariate correlations

DNA methylation and mRNA expression data from 379 HCC patients in a TCGA cohort were retrieved and the correlations between them were analysed using bivariate correlations. Among the 20 genes that are poorly studied in HCC (Table 1), DNA methylation data of *CLEC1B* and *SRPX* were not available. DNA methylation was negatively correlated with the mRNA expression for eight genes, *ACSM3*, *INTS8*, *LCAT*, *MT1X*, *CRHBP*, *MARCO*, *PAMR1*, and *VIPR1*. In particular, high methylation of the first four genes was significantly correlated with lower mRNA expression (Fig. 7), indicating that the expression of these genes in HCC might be regulated by DNA methylation.

## Discussion

Cancer is frequently considered to be a disease of the cell cycle because alterations in different families of cell cycle regulators cooperate in tumour development. Molecular analysis of human tumours has shown that cell cycle regulators are frequently mutated in human neoplasms, underscoring the importance of maintaining cell cycle commitment in the prevention of human cancer [26]. Abnormal expression of cell cycle controllers, particularly G1/S-phase transition, is often implicated in the pathogenesis of most human cancers, including HCC. For example, vaccinia-related kinase 1 promotes HCC by controlling the levels of cell cycle regulators associated with G1/S transition [27]. In this study, 80 genes that were significantly dysregulated in HCC were identified based on four independent microarrays covering a total of 386 cases of hepatocellular carcinoma and 327 cases of normal liver tissues (Fig. 1), and biological process annotation of these genes revealed that 17 of these genes were implicated in cell cycle functions (Additional file 1: Table S1). These results suggested that these genes might contribute to cancer progression and development in HCC at least in part through regulation of the cell cycle.

Twenty-seven genes were further identified to be consistently dysregulated in all four microarrays by at least 2-fold (Table 1). The expression of eight of these genes (*TBCE*, *INTS8*, *VIPR1*, *CLEC4M*, *MARCO*, *DNASE1L3*, *CRHBP*, and *FCN2*) was confirmed in 11 tissues of HCC patients compared with matched paracancerous tissues by RT-qPCR (Fig. 2a). Seven of the 27 genes (*UBE2C*, *PTTG1*, *CAP2*, *TOP2A*, *GPC3*, *EGR1*, and *NAT2*) have been well studied in HCC (Table 1). For example, GPC3 plays critical roles in cell proliferation and invasion through the induction of apoptosis [28] and is a biomarker for diagnosis [29] and recurrence [30]. Protein/gene-protein/gene interaction analyses indicated that these 27 proteins/genes strongly interacted with each other, and 10 of them interacted with at least half of all the genes (Additional file 2: Figure S2). Moreover, six of these genes were related to the cell cycle in HCC (Additional file 1: Table S1). Together, these results indicate that the genes identified in this study might play crucial roles in HCC progression, probably functioning as a group.

Biomarkers not only have prognostic implications, but are also helpful for measurement of treatment responses and surveillance for tumour recurrence and for guiding clinical decisions [31]. Thus, prognostic biomarkers for HCC patients are necessary and crucial, and there is an ongoing search for predictive biomarkers. In this study, a group of genes associated with DFS and OS (Table 2) were identified in 157 HCC patients. Among these genes, low expression of *ACSM3* and *CXCL14* was associated with poor DFS, low expression of *CRHBP*, *DNASE1L3*, *FCN2*, *MT1X*, and *VIPR1* was associated with poor OS (Fig. 3, Table 2), high expression of *INTS8* was associated with poor DFS and OS, and low expression of *LCAT*, *MARCO*, and *PAMR1* was associated with poor DFS and OS (Fig. 4, Table 2). Furthermore, *DNASE1L3* and *INTS8* were identified as independent risk prognostic factors for OS (Table 3). There are few reports of the association of these genes with prognosis in HCC or in other cancers. Previous studies indicate that downregulation of *CXCL14* is associated with prognosis in gastric cancer patients [32], *MT1X* may aid in the prognostic discrimination of oral squamous cell carcinoma cases [33], and *MARCO* expression is associated with breast cancer survival and risk of recurrence [34].

Twenty genes that have been less studied in HCC (Table 1) were further evaluated to predict their potential roles in HCC progression. Coremine medical mining suggested that most of those genes were associated with diagnosis, prognosis, drug resistance, recurrence, metastasis, and invasiveness. In particular, 13, 14, and 15 genes were potentially associated with prognosis, metastasis, and diagnosis in HCC, respectively (Fig. 5). The association of these genes with prognosis appears to have clinical importance, as 11 genes were shown to be associated with DFS or/and OS (Table 2, Fig. 3 & 4). The role of these genes in metastasis was further confirmed by gene expression analysis, which showed that five genes were significantly dysregulated in HCC with venous metastasis, portal vein tumour thrombus metastasis, or intrahepatic spread metastasis, compared with the appropriate controls. Specifically, *LCAT* was highly expressed in HCC patients with venous metastasis and patients with portal vein tumour thrombus metastasis, and *SRPX* was lowly expressed in HCC patients with venous metastasis and patients with intrahepatic spread metastasis (Fig. 6), suggesting that these two genes might be closely related to HCC metastasis. There are few studies on *LCAT* and *SRPX* in cancer metastasis, with only one reported that *SRPX* is upregulated in gastric cancer cells after depletion of TWIST, which promoted the epithelial-mesenchymal transition that occurs during the initial steps of tumour metastasis [35].

*INTS8* encodes a subunit of the integrator complex that is involved in the cleavage of small nuclear RNAs, and its association with cancer is poorly understood. Limited studies indicate that *INTS8* contains mutations in peripheral T cell lymphoma compared with non-malignant samples from 12 patients [36], and a combination of *INTS8* with *SULF1*, *ATP6V1C1*, and *GPR172A* can be used to discriminate gastric carcinomas from adjacent noncancerous tissues [37]. In this study, we found that, potentially regulated by demethylation (Fig. 7), *INTS8* was significantly and consistently upregulated at least 2.115-fold in HCC according to four independent microarrays (Fig. 1; Table 1) and that *INTS8* mRNA was upregulated 2.06-fold on average in 11 tissues of HCC patients compared with corresponding paracancerous tissues, with a similar expression profile at the protein level (Fig. 2). Based on the clinical importance analysis of 157 HCC patients in a TCGA cohort, we found that high expression of *INTS8* was associated with poor DFS and OS (Fig. 4, Table 2), and was an independent risk prognostic factor for OS (Table 3). Moreover, high expression of *INTS8* was associated with metastatic tumours and late stage (Table 4), and with younger HCC patients (<65 years old) (Table 4). In addition, text mining indicated that *INTS8* was closely related with metastasis, invasiveness, and diagnosis (Fig. 5). The above results strongly indicate that this gene is indeed upregulated in HCC, where it might play crucial roles in HCC cancer progression and development, and is a potential biomarker for diagnosis and, in particular, prognosis.

## Conclusion

In summary, by means of data retrieved from six independent microarrays, RT-qPCR and western blotting detection in 11 pairs of tissues, clinical importance analyses in a cohort of 157 patients, and bioinformatics analyses including biological process annotation, protein interaction and text mining, we have identified a group of genes that are significantly dysregulated in HCC and might be associated with cancer progression, development, and, in particular, prognosis. These genes could be potential therapeutic targets for HCC treatment, and might be useful biomarkers for diagnosis and prognosis.

## Additional files

Additional file 1: Table S1. Biological process and cellular component annotation of the 80 genes associated with HCC development and progression by DAVID online tool. (PDF 167 kb) Additional file 2: Figure S2. Protein/gene-protein/gene interaction network of the 27 genes that were stably and consistently dysregulated in 386 cases of hepatocellular carcinoma compared with 327 cases of normal liver tissue according to the four independent microarrays retrieved from the Oncomine database. (PDF 306 kb)
