# Supplementary material for: Microarray-based identification of genes associated with cancer progression and prognosis in hepatocellular carcinoma
Source: J Exp Clin Cancer Res. 2016 Aug 27;35(1):127. doi: 10.1186/s13046-016-0403-2 (PMC5002170; doi:10.1186/s13046-016-0403-2)
Supplement: Additional file 1: Table S1. — Biological process and cellular component annotation of the 80 genes associated with HCC development and progression by DAVID online tool. (PDF 167 kb) [file 13046_2016_403_MOESM1_ESM.pdf]

Discovery of microarray-identified genes correlates with development and prognosis of human hepatocellular carcinomas

Fuqiang Yin [1,2 #], Lipei Shu [3 #], Xia Liu [4 #], Ting Li [1], Tao Peng [3], Yueli Nan [5], Shu Li [5], Xiaoyun Zeng [2,5 *], Xiaoqiang Qiu [5 *]

[1] Medical Scientific Research Centre, Guangxi Medical University, Nanning, Guangxi 530021, P.R. China

[2] Key Laboratory of High-Incidence-Tumor Prevention and Treatment (Guangxi Medical University), Ministry of Education, Nanning, Guangxi 530021, P.R. China

[3] The First Affiliated Hospital, Guangxi Medical University, Nanning, Guangxi 530021, P.R. China

[4] Centre for Translational Medicine, Guangxi Medical University, Nanning, Guangxi 530021, P.R. China

[5] School of Public Health, Guangxi Medical University, Nanning, Guangxi 530021, P.R. China

# Contributed equally to this work

* Corresponding authors:

Prof. Xiaoyun Zeng, MD, PhD., School of Public Health, Guangxi Medical University, Nanning, Guangxi 530021, P.R. China. E-mail: zxyxjw@21cn.com

Prof. Xiaoqiang Qiu, MD, PhD., School of Public Health, Guangxi Medical University, 22 Shuangyong Road, Nanning, Guangxi 530021, P.R. China. E-mail: xqqiu9999@sina.com

Supplementary Table S1. Biological process and cellular component annotation of the 80 genes associated with HCC development and progression by DAVID online tool

Most highly enriched		P value	No. of genes	
Cell cycle-related (Biological processes)	Mitotic cell cycle	2.09E-06	12	17 genes in total (*AURKA*, *CAP2*, *CDK1*, *CDKN3*, *CKS1B*, *GMNN*, *KPNA2*, *NUSAP1*, *PRC1*, *PSMD4*, *PTTG1*, *RACGAP1*, *RAD21*, *TBCE*, *TOP2A*, *UBE2C*, *ZWINT*)
	Cell cycle process	2.15E-05	13	
	Cell cycle	2.53E-05	15	
	Cell division	1.10E-04	9	
	Cell cycle phase	2.15E-04	10	
	M phase	2.33E-04	9	
	Chromosome segregation	7.26E-04	5	
	Nuclear division	8.16E-04	7	
	Mitosis	8.16E-04	7	
	Microtubule cytoskeleton organisation	8.51E-04	6	
	M phase of mitotic cell cycle	8.97E-04	7	
	Organelle fission	0.001005	7	
	Spindle organisation	0.001482	4	
	Microtubule-based process	0.001675	7	
	Cytoskeleton organisation	0.006138	8	
	Regulation of cell cycle	0.006282	7	
Microtubule cytoskeleton-	Microtubule cytoskeleton	1.73E-04	11	14 genes in total (*AURKA*, *CCT3*, *CDK1*,
	Spindle	5.35E-04	6	

related (Cellular component)	Cytoskeletal component	0.003506	12	*ENAH*, *FCN2*, *HSPB1*, *NDRG2*, *NUSAP1*, *PEA15*, *PRC1*, *PSMB4*, *RACGAP1*, *TBCE*, *TOP2A*)
	Spindle microtubule	0.007579	3	
	Cytoskeleton	0.008458	14	
