# Supplementary material for: Microarray-based identification of genes associated with cancer progression and prognosis in hepatocellular carcinoma
Source: J Exp Clin Cancer Res. 2016 Aug 27;35(1):127. doi: 10.1186/s13046-016-0403-2 (PMC5002170; doi:10.1186/s13046-016-0403-2)
Supplement: Additional file 2: Figure S2. — Protein/gene-protein/gene interaction network of the 27 genes that were stably and consistently dysregulated in 386 cases of hepatocellular carcinoma compared with 327 cases of normal liver tissue according to the four independent microarrays retrieved from the Oncomine database. (PDF 306 kb) [file 13046_2016_403_MOESM2_ESM.pdf]

Discovery of microarray-identified genes correlates with development and prognosis of human hepatocellular carcinomas

Fuqiang Yin [1,2 #], Lipei Shu [3 #], Xia Liu [4 #], Ting Li [1], Tao Peng [3], Yueli Nan [5], Shu Li [5], Xiaoyun Zeng [2,5 *], Xiaoqiang Qiu [5 *]

[1] Medical Scientific Research Centre, Guangxi Medical University, Nanning, Guangxi 530021, P.R. China

[2] Key Laboratory of High-Incidence-Tumor Prevention and Treatment (Guangxi Medical University), Ministry of Education, Nanning, Guangxi 530021, P.R. China

[3] The First Affiliated Hospital, Guangxi Medical University, Nanning, Guangxi 530021, P.R. China

[4] Centre for Translational Medicine, Guangxi Medical University, Nanning, Guangxi 530021, P.R. China

[5] School of Public Health, Guangxi Medical University, Nanning, Guangxi 530021, P.R. China

# Contributed equally to this work

* Corresponding authors:

Prof. Xiaoyun Zeng, MD, PhD., School of Public Health, Guangxi Medical University, Nanning, Guangxi 530021, P.R. China. E-mail: zxyxjw@21cn.com

Prof. Xiaoqiang Qiu, MD, PhD., School of Public Health, Guangxi Medical University, 22 Shuangyong Road, Nanning, Guangxi 530021, P.R. China. E-mail: xqqiu9999@sina.com

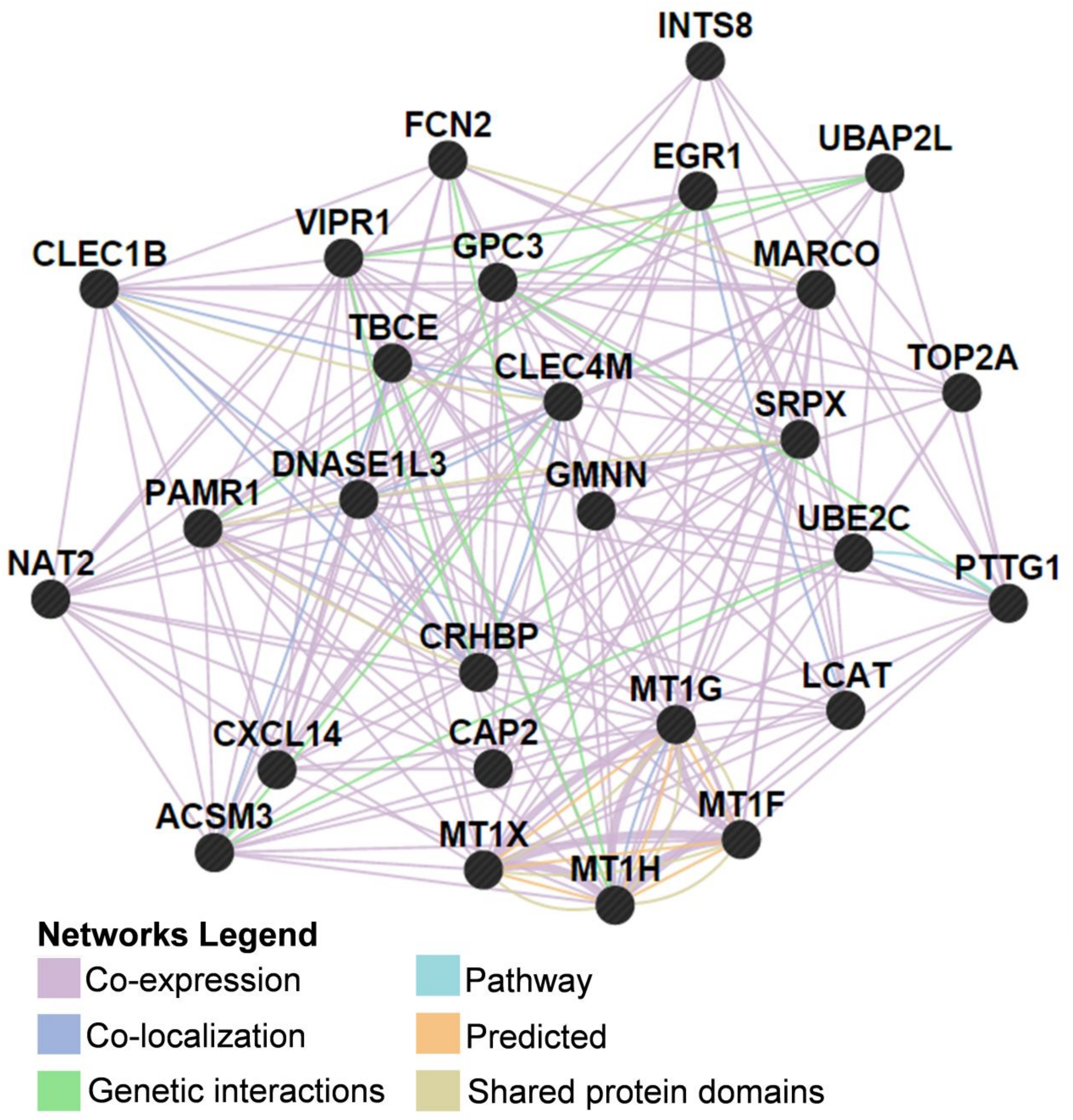

Supplementary Figure S2. Protein/gene-protein/gene interaction network of the 27 genes that were stably and consistently dysregulated in 386 cases of hepatocellular carcinoma compared with 327 cases of normal liver tissue according to the four independent microarrays retrieved from the Oncomine database. The network was generated using GeneMANIA tool. The interactions are indicated by the networks legend.
